# Urogenital Microbiota:Potentially Important Determinant of PD-L1 Expression in Male Patients with Non-muscle Invasive Bladder Cancer

**DOI:** 10.1186/s12866-021-02407-8

**Published:** 2022-01-04

**Authors:** Chunxiao Chen, Zehai Huang, Pengcheng Huang, Kun Li, Jiarong Zeng, Yuehui Wen, Biao Li, Jie Zhao, Peng Wu

**Affiliations:** 1 Department of Urology, Nanfang Hospital, Southern Medical University, Guangzhou, China; 2 The third hospital of mianyang, Sichuan Province, China; 3 Department of Urology, Meizhou People’s Hospital, Meizhou, China; 4 Meizhou hospital of TCM, Meizhou, China; 5 School of Pharmaceutical Sciences, Southern Medical University, Guangzhou, China; 6 Clinical Microbiota Center, Nanfang Hospital, Southern Medical University, Guangzhou, China

**Keywords:** Bladder cancer, Urogenital tract, Microbiota, PD-L1, Immune escape

## Abstract

**Background:**

Urogenital microbiota may be associated with the recurrence of bladder cancer, but the underlying mechanism remains unclear. The notion that microbiota can upregulate PD-L1 expression in certain epithelial tumors to promote immune escape has been demonstrated. Thus, we hypothesized that the urogenital microbiota may be involved in the recurrence and progression of non-muscle invasive bladder cancer (NMIBC) by upregulating the PD-L1 expression. To test this hypothesis, we investigated the relationship between urogenital microbial community and PD-L1 expression in male patients with NMIBC.

**Results:**

16S rRNA gene sequencing was performed to analyse the composition of urogenital microbiota, and the expression of PD-L1 in cancerous tissues was detected by immunohistochemistry. The subjects (aged 43–79 years) were divided into PD-L1-positive group (Group P, n = 9) and PD-L1-negative group (Group N, n = 19) respectively based on their PD-L1 immunohistochemical results. No statistically significant differences were found in the demographic characteristics between group P and N. We observed that group P exhibited higher species richness (based on Observed species and Ace index, both *P* < 0.05). Furthermore, subgroup analysis showed that the increase in number of PD-L1 positive cells was accompanied by increased richness of urogenital microbiota. Significantly different composition of urogenital microbiota was found between group P and group N (based on weighted Unifrac and unweighted Unifrac distances metric, both *P* < 0.05). Enrichment of some bacterial genera (e.g., *Leptotrichia*, *Roseomonas*, and *Propionibacterium*) and decrease of some bacterial genera (e.g., *Prevotella* and *Massilia*) were observed in group P as compared with group N. These findings indicated that these genera may affect the expression of PD-L1 through some mechanisms to be studied.

**Conclusion:**

Our study provided for the first time an overview of the association between urogenital microbiota and PD-L1 expression in male patients with NMIBC, indicating that urogenital microbiota was an important determinant of PD-L1 expression in male NMIBC patients.

**Supplementary Information:**

The online version contains supplementary material available at 10.1186/s12866-021-02407-8.

## Background

Bladder cancer, with an estimated 549,000 new cases and 200,000 deaths according to a 2018 report on the incidence and mortality of 36 cancers in 185 countries, ranked 10th among the most common malignancies worldwide [1]. And about 75% of bladder cancers are non-muscle invasive bladder cancer (NMIBC) at initial presentations, among which, Ta and T1 lesions are generally characterized with a good prognosis and low lethal potential [2], but, their 5-years recurrent rates are 50–70% after initial treatments, and 10–30% of which will even progress into muscle-invading tumor [3]. Researchers have long been studying the recurrence causes of non-muscle invasive bladder cancer, unfortunately, the exact causes and mechanisms remain unclear.

Some studies have shown that immune escape might be involved in the recurrence and progression of bladder cancer via the PD-L1/PD1 pathway [4, 5]. PD-L1 (B7H1) is a member of the B7 family of costimulatory molecules. It mainly promotes apoptosis by combining with programmed death receptors (PD1) expressed on the surface of T cells and B cells, thereby suppressing host immune function and achieving tumor immune escape [6, 7]. Hurwitz ME et al. reported that PD-L1 expression appears to increase as bladder cancer recurs [5]. The recurrence and progression rates of NMIBC are higher in patients with positive PD-L1 expression than that in patients with negative PD-L1 expression. And various factors and mechanisms are involved in the regulation of PD-L1 expression, such as IFNγ and microbiota [8–13]. Some researches have demonstrated that microbiota can upregulate the expression of PD-L1 in certain epithelial tumors and allergic diseases to promote immune escape or immune tolerance [11–13]. But, whether the microbiota in urogenital tract have the similar impact on PD-L1 expression in NMIBC remains unknown.

The human microbiota is the full collection of microbes (bacteria, fungi, parasites, viruses, etc.) that inhabit the epithelial barrier surfaces of our body [14], the microbiota data in this study were obtained by analyzing the mid-stream voided urines, thus the term “urogenital microbiota” was used [15]. In recent years, the relationship between urogenital microbiota and bladder cancer has received widespread attention. It is reported that tobacco smoking, carcinogen exposure and Schistosomiasis are the common predisposing factors of bladder cancer [16]. Besides, an association between chronic bladder infection with *Schistosoma haematobium* and the subsequent development of bladder squamous cell carcinoma in both men and women has been long-recognized [17], while Hicks RM et al. reported that strains of bacteria may contribute to schistosomiasis-induced bladder cancer by mediating the formation of N-nitrosamines [18, 19]. Bacillus Calmette-Guerin (BCG), the *Mycobacterium bovis*-derived vaccine strain for tuberculosis, is widely used to prevent recurrence of bladder cancer by direct bladder instillation [20]. Since commensal microorganisms are also present in the bladder, they may potentially interact with BCG, influencing the efficacy of BCG [21]. Our previous study found that *Herbaspirillum*, *Porphyrobacter* and *Bacteroides* are enriched in bladder cancer patients with a high risk of recurrence and progression, which suggests that these genera might be associated with the recurrence of bladder cancer [22]. Zitvogel et al. reported that oral administration of *Lactobacillus* after removal of the bladder tumor could reduce the probability of recurrence [23], which added evidence to the relationship between microbiota and bladder cancer recurrence. The composition of urogenital microbiota is closely related to tobacco smoking, which indicates tobacco smoking may also promote the occurrence and development of bladder cancer by changing urogenital microbiota [24]. However, the underlying mechanism of urogenital microbiota affecting the recurrence of NMIBC remains to be further studied. The important roles of microbiota in multiple systems of the human body have been proven, including the immune system [25]. Therefore, based on the potential relationship between microbiota and PD-L1 expression, we hypothesized that the urogenital microbiota may be involved in the recurrence and progression of NMIBC by up-regulating PD-L1 expression.

In this study, we mainly aimed to assess the association between urogenital microbiota and PD-L1 expression in male patients with NMIBC and to screen for some potential taxa related to PD-L1 expression. The results can serve as a resource for further researches to gain a better understanding of the role of urogenital microbiota in tumor recurrence and identify candidate biomarkers for the application of PD1 or PD-L1 blockers in bladder cancer.

## Results

### PD-L1 Expression on Tumor Cell Membrane or Immune Cells

**Fig. 1:**
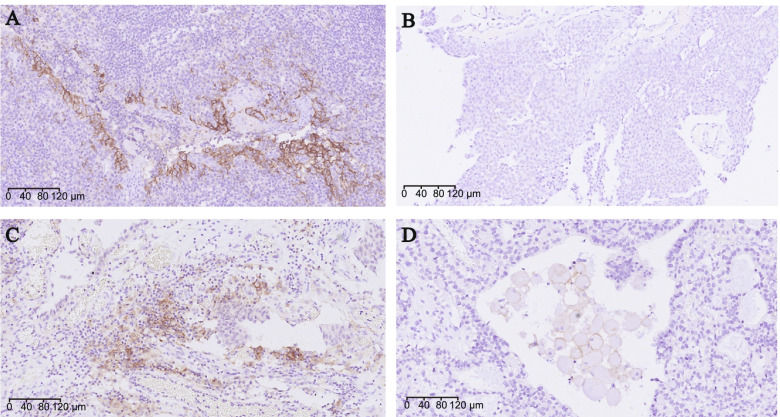
PD-L1 expression in formalin-fixed paraffin-embedded tissue samples stained with anti-PD-L1 antibody (405.9A11). Positive control with tonsil tissue for PD-L1 expression is presented in panel **(A)**. In panel **(B)**, both tumor cells and immune cells are negative for PD-L1. Positive membranous staining in tumor cells and immune cells are presented in panels **(C)** and **(D)**, respectively

16S rRNA gene sequence data of 28 male patients with NMIBC were obtained, and their tumor samples were used to detect PD-L1 expression on tumor cells or immune cells. Among them, the tumor stage of 13 patients was TaN0M0, while 15 patients was T1N0M0. Overall, PD-L1 expression was negative in 19 patients (67.9%) and positive in 9 patients (32.1%, including 6 patients with PD-L1 expression on tumor cells and 3 patients on immune cells). And PD-L1 did not appear in tumor cells and immune cells simultaneously in PD-L1 positive specimens. Out of the 28 male patients with NMIBC, the intensity of PD-L1 was assessed and recorded as absent (0) in 19 patients (67.9%), mild (1+) in 6 patients (21.4%), moderate (2+) in 1 patient (3.6%), and severe (3+) in 2 patients (7.1%). Representative immunohistochemical staining results are shown in Fig. 1.

### Demographic Characteristics of Subjects

A total of 31 mid-stream urine specimens were analyzed, including 11 from male NMIBC patients with positive PD-L1 expression and 20 from male NMIBC patients with negative PD-L1 expression, while 2 specimens in PD-L1-positive group and 1 specimen in PD-L1-negative group were excluded for too little sequencing reads. Finally, 28 male patients with NMIBC based on their PD-L1 immunohistochemical results were divided into PD-L1-positive group (group P) and PD-L1-negative group (group N), respectively. No statistically significant differences were found in the demographic characteristics between group P and N (**Table** **1**).

**Table 1:** Comparisons of demographic characteristics between PD-L1-positive (group P) and PD-L1-negative groups (group N)

Demographic characteristics	Group P (*n*=9)	Group N (*n*=19)	*P*-value
Age (y)	67.11 (11.48)^a^	62.05 (9.79)^a^	0.238
Weight (kg)	63.92 (9.03)^a^	64.79 (7.96)^a^	0.798
Height (m)	1.66 (0.06)^a^	1.68 (0.05)^a^	0.270
BMI (kg/m^2^)	23.16 (2.00)^a^	22.89 (2.76)^a^	0.794
Drinking history	11.11% (1/9)	21.05% (4/19)	1.000
Hypertension	44.44% (4/9)	31.58% (6/19)	0.677
Diabetes	33.33% (3/9)	10.53% (2/19)	0.290
Hyperlipemia	22.22% (2/9)	0.00% (0/19)	0.095
CHD	22.22% (2/9)	10.53% (2/19)	0.574
FHC	0.00% (0/9)	5.26% (1/19)	1.000
Smoking index	200 (0,1500)	400 (0,800)	0.841
Percentage of smokers	55.56% (5/9)	73.68% (14/19)	0.407
Percentage of multiple tumors	22.22% (2/9)	52.63% (10/19)	0.223
Percentage of hypergrading	77.78% (7/9)	42.11% (8/19)	0.114
EORTC_R	4.44 (2.79)^a^	4.37 (2.83)^a^	0.947
Percentage of HER	55.56% (5/9)	42.11% (8/19)	0.689
EORTC_P	8.89 (4.78)^a^	6.63 (5.36)^a^	0.292
Percentage of HEP	66.67% (6/9)	47.37% (9/19)	0.435
Percentage of high invasive risk	88.89% (8/9)	63.16% (12/19)	0.230
Percentage of T1N0M0	77.78% (7/9)	42.11% (8/19)	0.114

Data are presented as mean (SD) or median (first quartile to the third quartile) for continuous variables or n (%) for counting data

^a^Indicates that the datum is subject to normal distribution and homogeneity of variance test and is presented as mean (SD). Multiple tumors refer to the number of bladder tumor ≥ 2. Abbreviations: BMI, body mass index; CHD, coronary atherosclerotic heart disease; FHC, family history of cancer; EORTC, European Organization Research and Treatment of Cancer Scoring system, _R refer to recurrence and _P refer to progression; HER, recurrence score of EORTC ≥ 5; HEP, progression score of EORTC ≥ 7

### Sequencing Data, Alpha, Beta Diversity and Alpha Rarefaction Curves

**Fig. 2:**
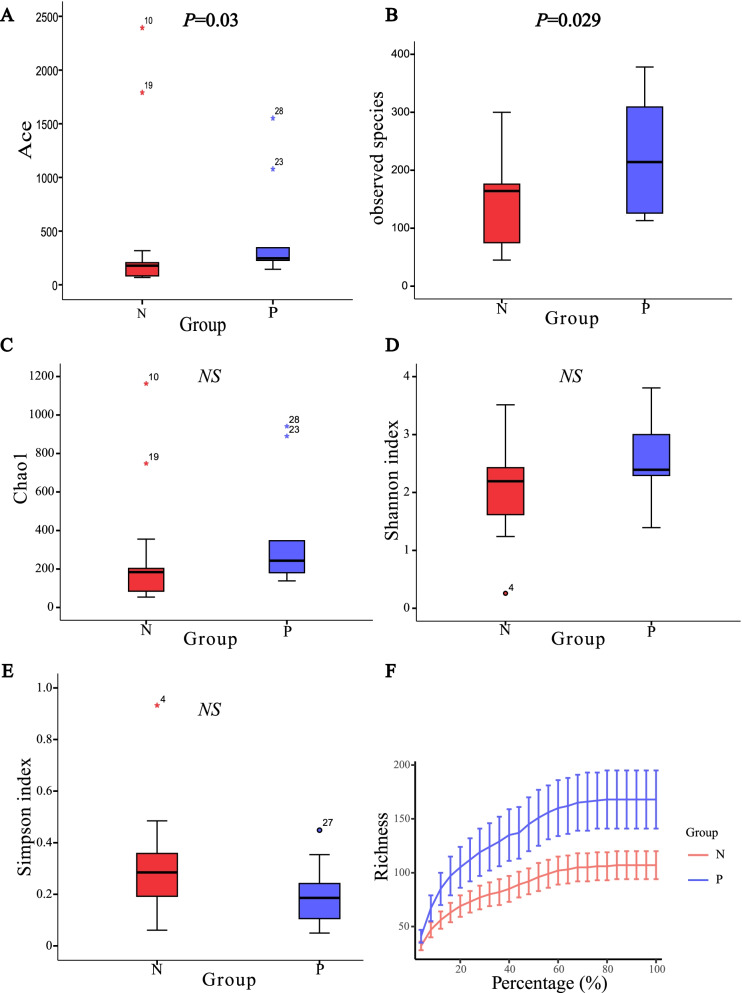
Alpha Diversity and Alpha rarefaction curves for PD-L1-positive (group P) and PD-L1-negative groups (group N). Ace index **(A)**, observed species **(B)**, Chao1 index **(C)**, Shannon index **(D)** and Simpson index **(E)** were used for the assessment of alpha diversity between group P and group N. Rarefaction curves show that the Richness of group P was much higher than that of group N **(F)**

Reads that contain over 50% of base with quality value less than 20 or that contain over 65% of base with quality value less than 30 were removed. A total of 962,452 reads were obtained from 28 samples after the removal of primer and chimera. The median number of reads in PD-L1 positive group was 26,335, and in the PD-L1 negative group was 34,952 (*P* = 0.676). And the reads were classified into 2,124 OTUs which were used for downstream analysis. More OTUs were identified in urine samples from group P, with an average of 215 OTUs per sample in group P and 141 OTUs per sample in group N (*P* = 0.029). The number of reads and OTUs for each sample are shown in Supplementary Table S1. The Ace index, Observed species and Rarefaction curves all shown that the urogenital microbiota in group P was much richer than that in group N. (*P* < 0.05; Figs. 2A, B and 2F). While no difference was found in the Chao1 index (Fig. 2C), Shannon index (Fig. 2D) and Simpson index (Fig. 2E) between group P and group N.

In order to assess the relationship between PD-L1 expression intensity and urogenital microbiota, we divided group P into group C (with mild PD-L1 expression) and group D (with moderate or severe PD-L1 expression) based on the expression intensity of PD-L1. However, no statistically significant difference in the bacterial richness was found between the two groups (Supplementary Figure S1). Among PD-L1 positive group, urogenital microbiota of patients with ≥ 5% tumor cells membrane staining had higher richness than that of patients with < 5% (*P* <0.05; Supplementary Figure S2).

**Fig. 3:**
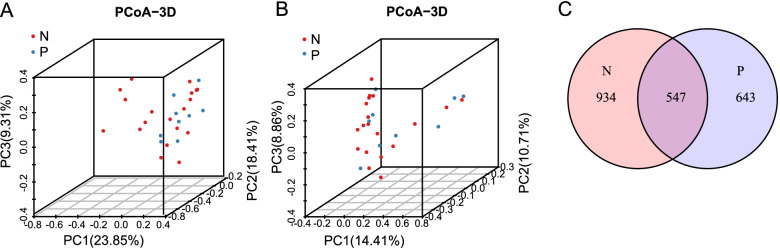
The composition of urogenital microbiota in group P differed from that in group N. Principal coordinate analysis (PCoA) of weighted UniFrac distance **(A)** and unweighted UniFrac distance **(B)** show that the composition of urogenital microbiota in group P differed from that in group N (*P* < 0.05 and *P* = 0.047 for weighted UniFrac and unweighted UniFrac distances, respectively). Venn diagrams **(C)** depict different OTU compositions between group N and group P

To measure the similarity of urogenital microbial communities between group P and group N, we performed the unconstrained principal coordinate analysis (PCoA) based on weighted UniFrac and unweighted UniFrac distance metrics. The PCoA plots revealed that the composition of urogenital microbiota in group P differed from that in group N (*P* < 0.05 and *P* = 0.047 for weighted UniFrac and unweighted UniFrac distances, respectively. Figs. 3A, B). Besides, we detected that 643 OTUs were enriched in group P, while 934 OTUs in group N, and 547 OTUs were shared between the two groups (Fig. 3C).

### Relative Abundance of Urogenital Bacteria in group P and group N

**Fig. 4:**
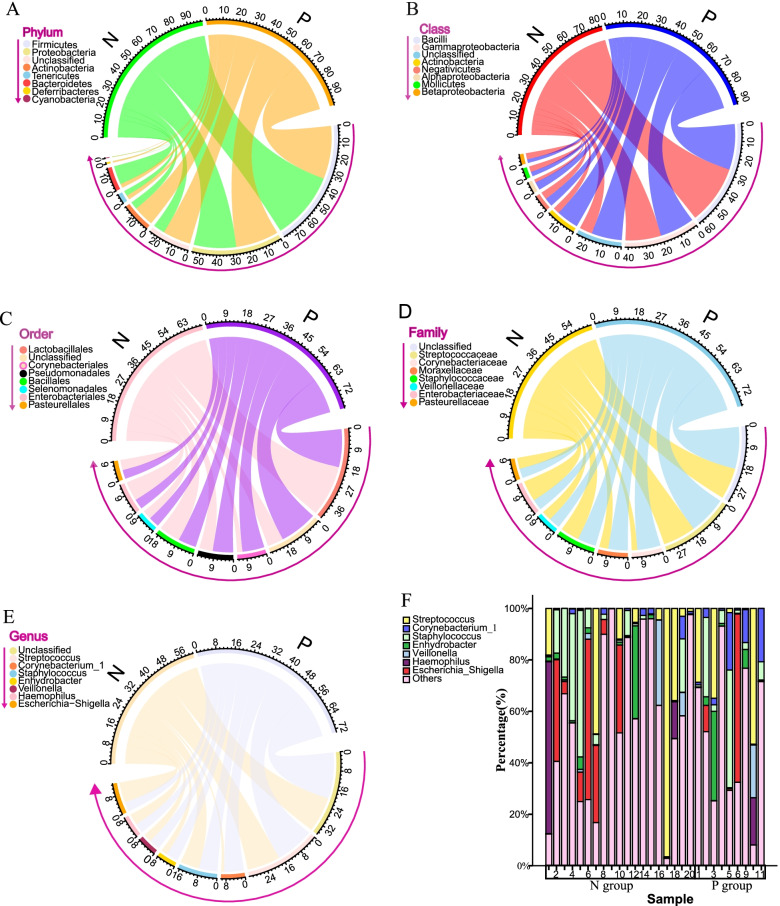
Bacterial relative abundance in PD-L1 positive group and PD-L1 negative group. Average distributions of 8 major taxa are represented by circlize graphs at the level of Phylum **(A)**, Class **(B)**, Order **(C)**, Family **(D)** and Genus **(E)**. Each color represents a bacterial taxon which is displayed sequentially in the direction indicated by the arrow and the width of a colored ribbon represents the relative abundance of that organism within the sample. In percentage bar chart **(F)**, each bar represents a subject and each colored box, a bacterial taxon. The height of a colored box represents the relative abundance of that organism within the sample

At phylum level, the urogenital microbiota was dominated by Firmicutes (22.8% in group P, 34.4% in group N) and Proteobacteria (21.1% in group P, 25.8% in group N), followed by Actinobacteria (6.6% in group P, 4.0% in group N) and Bacteroidetes (1.1% in group P, 9.7% in group N) (Table 2, Fig. 4A). The urogenital microbial compositions of different groups at class, order, family and genus levels were exhibited in Figs. 4B–E. The genera compositions of all samples were exhibited in Fig. 4F. Though the relative abundance of so many microbes at various taxonomic levels differed between group P and group N, only five taxa including Bacteroidetes (*P* = 0.017), Bacteroidia (*P* = 0.022), Bacteroidales (*P* = 0.025), Prevotellaceae (*P* = 0.028) and *Prevotella* (*P* = 0.04) were present with higher relative abundance in group N and one genus called *Leptotrichia* had higher abundance in group P (*P* = 0.001), when the Metastats algorithm was used for comparison of the relative abundance of bacteria and the relative abundance threshold of microbes was set at 0.1% (Table 2).

**Table 2:** Comparison of relative abundance of urogenital microbiota between PD-L1 positive (group P) and PD-L1 negative groups (group N)

Taxa		Group N	Group P	*P*-value
Phylum	Firmicutes Proteobacteria Bacteroidetes Actinobacteria	34.438 25.756 9.662 4.012	22.828 21.077 1.130 6.631	Ns Ns 0.017 Ns
Class	Bacteroidia Betaproteobacteria Negativicutes Actinobacteria Bacilli Unclassified Gammaproteobacteria Alphaproteobacteria	9.334 4.557 3.230 3.085 27.572 22.313 19.051 1.765	0.856 2.234 2.353 6.488 19.562 44.950 16.725 2.012	0.022 Ns Ns Ns Ns Ns Ns Ns
Order	Enterobacteriales Bacteroidales Pseudomonadales Pasteurellales Burkholderiales Selenomonadales Unclassified Lactobacillales Bacillales Corynebacteriales	9.744 9.316 5.051 3.973 3.477 3.226 23.076 16.826 10.619 1.928	6.043 0.853 6.358 1.995 2.042 2.352 46.121 11.731 7.522 4.426	Ns 0.025 Ns Ns Ns Ns Ns Ns Ns Ns
Family	Enterobacteriaceae Prevotellaceae Staphylococcaceae Unclassified Pasteurellaceae Veillonellaceae Moraxellaceae Comamonadaceae Pseudomonadaceae Streptococcaceae Corynebacteriaceae	8.580 7.980 7.876 31.822\ 3.966 2.603 2.510 2.452 2.335 10.553 1.788	6.001 0.761 7.288 46.981 1.995 2.347 5.147 1.804 1.117 10.664 4.296	Ns 0.028 Ns Ns Ns Ns Ns Ns Ns Ns Ns
Genus	*Escherichia-Shigella* *Prevotella* *Staphylococcus* *Unclassified* *Haemophilus* *Pseudomonas* *Streptococcus* *Delftia* *Veillonella* *Enhydrobacter* *Leptotrichia*	8.167 8.060 7.187 36.701 3.899 2.306 10.433 1.817 1.727 1.332 0.000	4.840 0.672 6.941 49.489 1.983 1.106 10.485 1.475 2.213 3.560 0.205	Ns 0.040 Ns Ns Ns Ns Ns Ns Ns Ns 0.001

Data were displayed as mean percentage; For taxa with *P*-value < 0.05, the taxa shown in the table are those with relative abundance greater than 0.1%. For taxa without significant difference, only taxa with relative abundance greater than 1% are shown in the table; Ns, not significant (based on *P* < 0.05)

### Specific Taxa Associated With PD-L1 Expression

**Fig. 5:**
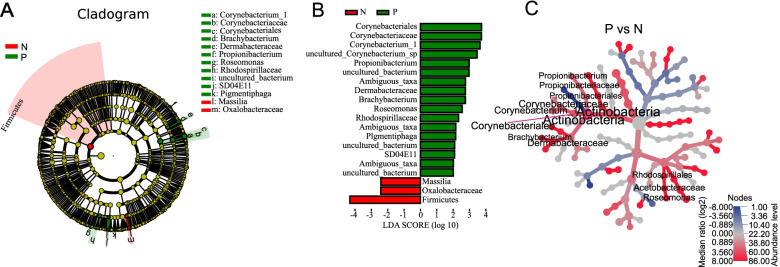
Specific taxa associated with PD-L1 expression. The urogenital microbial taxa associated with PD-L1 positivity (green) and PD-L1 negativity (red) are revealed in Cladogram (**A**). Association of specific microbial taxa with PD-L1 positive and PD-L1 negative group is exhibited by linear discriminant analysis effect size (LEfSe) (**B**). Green indicates taxa enriched in PD-L1 positive group and red indicates taxa enriched in PD-L1 negative group. Heat tree is used to illustrate the taxonomic differences between group N and group P (**C**). The color gradient and the size of node, edge and label are based on the log2 ratio of median abundance. Blue and red indicate that corresponding taxa are lower and higher, respectively, in group P as compared with group N

The specific microbial taxa associated with PD-L1 expression were identified by using LEfSe algorithm. The results showed that Corynebacteriale, Corynebacteriaceae, *Corynebacterium_1*, *uncultured_Corynebacterium_sp*, *Propionibacterium*, Dermabacteraceae, *Brachybacterium*, *Roseomonas*, Rhodospirillaceae, *Pigmentiphaga* were present at significantly higher compositional abundances in group P compared with group N, whereas the relative abundance of *Massilia*, Oxalobacteraceae, Firmicutes were higher in group N (Figs. 5A, B). Heat tree was used to illustrate the taxonomic differences between group N and group P (Fig. 5C). Similarly, in group P, *Roseomonas* was enriched in the group with higher PD-L1 expression (group F, PD-L1 ≥ 5%) and *Prevotella* had higher abundance in the group with lower PD-L1 expression (group O, 1% ≤ PD-L1 < 5%) (Supplementary Figure S3).

## Discussion

Over the past decade, the microbiota in different parts of the human body have been characterized and some microbiota-related mechanisms which can directly impact the human health have begun to be identified [26]. Developing diagnostics and therapeutics that utilize those connections between the microbiome and the human health is the next frontier for translational microbiome science [27]. However, the falsifiable hypothesis needs to be discovered through observational studies before performing a randomized validation experiment. In this exploratory study, we found the associations between urogenital microbiota and PD-L1 expression in patients with NMIBC and some genera related to PD-L1 expression which may be isolated by bacterial culture technique and experimentally verified for their roles in regulating the expression of PD-L1. The findings of this study can serve as a resource for further researches to gain a better understanding of the roles of urogenital microbiota in tumor recurrence and identify candidate biomarkers for the application of PD1 or PD-L1 blockers in bladder cancer.

The role of PD-L1/PD1 mediated immune escape in bladder cancer recurrence and progression has long been studied [4, 5], which has facilitated the development of clinical trials of PD-L1/PD1 inhibitors in NMIBC and the FDA’s approval of 5 PD-L1/PD1 inhibitors for the treatment of metastatic bladder cancer [28]. And in order to improve the response rate and reduce drug-related adverse reactions, patients with bladder cancer often receive PD-L1/PD1 inhibitors therapy depending on their PD-L1 status [29], which indicates the PD-L1 expression level is still one of the main factors that determine the efficacy of PD1/PD-L1 inhibitors. Therefore, exploring factors affecting the expression of PD-L1 may help improve the therapeutic effect of PD-L1/PD1 inhibitors. Groeger S et al. have reported that *Porphyromonas gingivalis* and its total membrane fraction can upregulate PD-L1 expression in vitro [11, 12]. In addition, microbiota could promote immune tolerance to allergens in neonates via PD-L1 [13]. And the concept that microbiota can modulate the clinical response to PD1/PD-L1 inhibitors has been confirmed in experimental studies [30, 31], though the underlying mechanisms are still unknown. Previous studies and ours have demonstrated the correlation between the microbiota and PD-L1 expression, suggesting that the microbiota may affect the therapeutic effect of PD1/PD-L1 inhibitors by regulating PD-L1 expression. If the roles of urogenital microbiota in PD-L1 expression would be confirmed in the future, urogenital microbiota might be a target for enhancing clinical responses to PD1/PD-L1 inhibitors in the treatment of bladder cancer or other urogenital tract tumors.

Increased microbial richness was observed both in muscle-invasive bladder cancer patients and NMIBC patients with a high risk of recurrence and progression [22], which was consistent with the results of Hai Bi group [32]. In present study, significantly higher bacterial richness in the PD-L1 positive group was found as well, which suggests that higher bacterial richness may be combined with PD-L1 or used as a substitute for PD-L1 to predict the recurrence or progression of NMIBC. Despite the inter-individual differences in the microbiota composition, the urogenital microbiota of individuals in the same group still have similarities, as shown in the PCoA analysis that clustered PD-L1 positive group and PD-L1 negative group separately (Figs. 3A and 3B), indicating the possibility of a common urogenital microbiota disorder associated with PD-L1 expression.

In our study, we found that the *Leptotrichia* was enriched in PD-L1 positive male patients with NMIBC and *Prevotella* was enriched in PD-L1 negative group. *Fusobacterium* and *Leptotrichia* are closely-related organisms. They have been isolated from periodontal lesions and a diversity of genitourinary and gastrointestinal abscesses, and have been suggested to be potential emerging pathogens [33]. Studies have demonstrated that *Leptotrichia* is involved in the occurrence and development of several carcinomas, such as gastric cancer, colon cancer, pancreatic cancer and etc [34–38]. Jang et al. reported IL-6 and IL-8 were strongly induced by *Leptotrichia wadei* [39], and IL6 is an inflammatory cytokine involved in various biological processes, including immune disorders and cancers [40, 41]. Chan et al. have demonstrated that Pro-inflammatory cytokines such as IFNγ and IL-6, could promote the expression of PD-L1 to achieve tumor immune escape [8, 42], which is consistent with our results. However, whether *Leptotrichia* can induce the expression of PD-L1 in bladder cancer and its related mechanisms need further confirmation in vivo and in vitro. As for *Prevotella*, Calcinotto A and collaborators recently reported that the abundance of *Prevotella histicola* and *Prevotella melaninogenica* in the guts of preclinical models and the humans or their colonization of the mouse guts are associated with decreased proinflammatory Th17 cells in the intestine and bone marrow, leading to delayed multiple myeloma progression [43]. On the contrary, *Prevotella heparinolytica* accelerated the progression of multiple myeloma in mice by inducing the local differentiation of Th17 cells that migrated to the bone marrow and supported plasma cells’ survival and *Prevotella* spp. could sustain the protumorigenic effect of dextran sodium sulfate by favoring Th17 and Th1 immunity in the colon as well [43, 44]. Taken together, data from malignancy patients and murine experimental models converge to support the perspective that *Prevotella* may participate in the progression of malignant tumors through Th17 cells, but different strains of *Prevotella* may play opposite roles.

*Roseomonas* and *Propionibacterium* were found in higher abundances in PD-L1 positive group than in PD-L1 negative group. *Roseomonas mucosa* may directly influence atopic dermatitis and provide clinical benefit through multiple mechanisms, including innate/adaptive immune balance [45]. While *Propionibacterium* strain, P. UF1, can safeguard against proinflammatory diseases by increasing the frequencies of colonic Th17 and Treg cells [46]. These studies, together with ours, indicate that these two genera may affect the course of human diseases by regulating host's immunity.

This study does have limitations. First of all, the causal relationship between the urogenital microbiota and PD-L1 expression can not be determined by this observational study, which requires further prospective studies and experiments in vivo and in vitro to verify. Secondly, although it is a far less invasive method than suprapubic aspiration or catheterization and therefore is suitable for daily practice, mid-stream urine specimens collected by the clean catch method may be contaminated by microbiota in the urethra or surrounding the urethral orifice. Last but not least, 16S rRNA gene sequencing may reliably characterize microbiota to the genus level but cannot identify bacteria well at species level and detect viruses or fungi. However, its low cost and easy sampling make 16S rRNA gene sequencing always a pilot in metagenome and other microbiota-related researches.

## Conclusions

This is the first study to profile urogenital microbiota associated with the expression of PD-L1 in male patients with NMIBC. Our study shows that urogenital microbiota is associated with PD-L1 expression in male patients with NMIBC, but the exact relationship remains to be further investigated. Further exploration of the underlying mechanisms to explain these associations will offer new prospects for researches on tumor recurrence and application of PD1 or PD-L1 blockers in bladder cancer.

## Methods

### Study design, subject recruitment and sample collection

The aim of this cross-sectional study was to characterize urogenital microbiota associated with PD-L1 expression in male patients with NMIBC. Urine specimens and cancerous tissues were collected from male patients with NMIBC who were admitted to Nanfang Hospital in China between March 2017 and June 2019. Eventually, twenty-eight subjects histologically confirmed as non-muscle invasive bladder cancer, aged from 43 to 79 years, were enrolled for the study. The urine specimens were collected prior to the prescription of antibiotics and operation for bladder cancer. Exclusion criteria for the participants included prior known sexually transmitted infection, current urinary tract infection (based on urine dipstick or standard cultivation) or antibiotic consumption within 1 month for any reason. All participants were required to finish a questionnaire to collect their demographic and medical information. Following the Medicine Institutional Review Board of Southern Medical University approval (ethical code: NFEC-2020-045) and the Declaration of Helsinki principles, all participants gave written informed consent for their information, urine and tissue collection with analysis for research purposes.

All urine specimens for urinalysis were obtained by the mid-stream clean catch method with the guidance of urotherapy nurses, then immediately shipped on cold pack to laboratory within an hour for centrifugation at 16,000 g for 10 min at 4°C. The precipitates were stored at −80 degrees Celsius until further processing.

### DNA Isolation and 16S rRNA Gene Sequencing

To avoid contamination, DNA was extracted using the DNeasy Blood and Tissue Kit following the cultured cells protocol provided by the manufacturer (Qiagen, Germany) in a laminar flow hood. The Nanodrop ND-1000 spectrophotometer (Thermo Electron Corporation, USA) was used for the measurement of extracted DNA concentration. 16S rDNA sequences of the genomic DNA isolated from the urine specimens was amplified with PCR using primer sets specific for V3-V4 regions (314F: 5′-NCCTACGGGNGGCWGCAG-3′; and 805R: 5′NGACTACHVGGGTATCTAATCC-3′) [32]. We added extraction negative controls (no urine) and PCR negative controls (no template) to evaluate the presence of contaminating sequences in reagents. Unincorporated nucleotides and primers were eliminated using the Qiaquick PCR purification kit (Qiagen, Valencia, USA) to obtain the final PCR purified products [47]. After normalization to equal DNA concentration, the purified samples were sequenced using the Illumina Miseq sequencer (Illumina, USA). The 16S rRNA gene sequence data reported in this paper have been deposited in the Genome Sequence Archive (Genomics, Proteomics & Bioinformatics 2017) in BIG Data Center (Nucleic Acids Res 2018), Beijing Institute of Genomics (BIG), Chinese Academy of Sciences, under accession numbers CRA002985, CRA002985 that are publicly accessible at https://bigd.big.ac.cn/gsa.

### Immunohistochemistry

Immunohistochemical detection of PD-L1 was performed on formalin-fixed paraffin-embedded tissues using a mouse monoclonal anti-PD-L1 antibody (405.9A11, dilution 1:200, Cell Signaling Technology) and following the protocol provided by the manufacturer [48]. For each sample, the membranous expression of PD-L1 in tumor cells or immune cells was determined and scored in blinded manner with respect to clinical data by two independent genitourinary pathologists. The tumor was considered positive for PD-L1 if ≥ 1% of tumor cells or immune cells had histologic evidence of plasma membrane staining. PD-L1 expression in tumor cells or immune cells was scored as the percentage of stained cells. And the intensity of PD-L1 was assessed and recorded as absent (0), mild (1+), moderate (2+) and severe (3+).

## Statistical Analysis

### Clinical Data Analysis

Normal distributions of the data were estimated with Shapiro–Wilk test and homoscedasticity of variances was checked using Levene test. The differences between groups were tested using Student’s t test for measurement data subject to normal distribution and homogeneity of variance and Fisher’s Exact Test for counting data. While Mann-Whitney U test was used for the statistical analysis of ranked data or measurement data that did not conform to normal distribution or homogeneity test of variance. Statistical analysis was performed using SPSS statistics version 24 (IBM corp, Amonk, NY, USA). All tests were two sided and *P* < 0.05 was considered statistically significant.

### Bioinformatics analysis

In order to obtain clean reads, the raw data of 16S rRNA gene sequences were filtered using QIIME [49] to eliminate reads with adapter pollution and low quality. Metadata are given in Supplementary Table S2. The paired-end reads were merged with their overlap using standardized bioinformatic pipeline to attain complete sequence which were clustered into operational taxonomic units (OTUs) with 97% similarity and the representative sequences were picked by UPARSE [50–52]. Subsequently, UCHIME method in VSEARCH (v2.3.4) was used for the detection and removal of chimera [53, 54]. The representative sequence from each clustered OTU was aligned to the SILVA database and the Greengenes database and classified with the RDP classifier to achieve species annotation [55–57].

Observed Species, Chao1, Ace, Shannon and Simpson indices were calculated using QIIME to evaluate alpha diversity. Among them, the Observed Species, Chao1 and Ace indices are mainly used to assess the species richness of samples, while Shannon and Simpson indices mirror both species richness and evenness. The difference of alpha diversity between groups was evaluated by Mann-Whitney U Test (group number = 2) or Kruskal–Wallis test (group number > 2) using SPSS statistics version 24 (IBM corp, Amonk, NY, USA) or R software. The weighted UniFrac and unweighted UniFrac distances were calculated for the comparison of Beta-diversity of different groups. Three-dimensional plots were generated based on Principal coordinate analysis (PCoA) of these distance matrices in QIIME for the visual clustering of the bacterial communities. To statistically support the differences in beta-diversity between groups, these distances of different groups were compared using Adonis test or ANOSIM test.

Average distribution of major taxa was represented by circlize graphs which were drawn with the circlize package in R. To detect significantly different bacteria between groups, the relative abundance of bacteria were compared using Metastats. Taxa summaries were input into Linear discriminant analysis effect size (LEfSe) via the Huttenhower Lab Galaxy Server for further screening of significantly different bacteria between groups at different taxonomic level [58]. In the settings of LEfSe, the significantly specific bacteria were identified using the Mann-Whitney U test, and their effect size were estimated via linear discriminant analysis (LDA) of which the threshold for discriminative features was 2.0. While heat tree was generated with the workflow provided by Chong et al. [59] and the use of MicrobiomeAnalyst (https://www.microbiomeanalyst.ca).

## Supplementary Information

**Additional file 1: Supplementary Table S1.**The number of reads and OTUs for each sample.

**Additional file 2: Supplementary Table S2.** The information of Metadata.

**Additional file 3: Figure S1.** Alpha Diversity for group C (with mild PD-L1 expression) and group D (with moderate or severe PD-L1 expression).observed species (A), Chao1 index (B), Ace index (C), Shannon index (D) and Simpson index (E) were used for the assessment of alpha diversity between group C and group D. We found that as the expression intensity of PD-L1 increased, the bacterial richness increased as well. Ns, not significant (based on P < 0.05).

**Additional file 4: Figure S2**. Alpha Diversity for group F (with ≥5%tumor cells membrane staining) and group O (with < 5% tumor cells membrane staining). observed species(A),Chao1 index (B),Ace index(C),Shannon index (D) and Simpson index (E) were used for the assessment of alpha diversity between group F and group O. Urogenital microbiota of group F had higher richness than that of group O. Ns, not significant (based on P < 0.05).

**Additional file 5: Figure S3.** Specific taxa associated with different PD-L1 expression levels. Association of specific microbial taxa with higher PD-L1 expression (group F, PD-L1≥5%) and lower PD-L1 expression (group O, 1%≤PD-L1<5%) is exhibited by linear discriminant analysis effect size (LEfSe).Green indicates taxa enriched in group O and red indicates taxa enriched in group F.

## Data Availability

The 16S rRNA gene sequence data reported in this paper have been deposited in the Genome Sequence Archive (Genomics, Proteomics & Bioinformatics 2017) in BIG Data Center (Nucleic Acids Res 2018), Beijing Institute of Genomics (BIG), Chinese Academy of Sciences, under accession numbers CRA002985, CRA002985 that are publicly accessible at https://bigd.big.ac.cn/gsa.

## Abbreviations

NMIBC: Non-muscle invasive bladder cancer
PD-L1: Programmed Cell Death 1 Ligand 1
PD1: Programmed Cell Death Protein 1
PCoA: Principal coordinate analysis
LEfSe: Linear discriminant analysis (LDA) effect size
PCR: Polymerase chain reaction
OTUs: Operational taxonomic units
QIIME: Quantitative Insights Into Microbial Ecology
