# Supplementary material for: Urogenital Microbiota:Potentially Important Determinant of PD-L1 Expression in Male Patients with Non-muscle Invasive Bladder Cancer
Source: BMC Microbiol. 2022 Jan 4;22:7. doi: 10.1186/s12866-021-02407-8 (PMC8725255; doi:10.1186/s12866-021-02407-8)
Supplement: Supplementary file 1 — Additional file 1: Supplementary Table S1.The number of reads and OTUs for each sample. [file 12866_2021_2407_MOESM1_ESM.docx]

**Supplementary Table S1.The number of reads and OTUs for each sample.**

| Sample name | reads number | OTUs number |
| --- | --- | --- |
| P9 | 61321 | 315 |
| P10 | 66364 | 126 |
| N14 | 43360 | 181 |
| N15 | 45124 | 164 |
| N16 | 53635 | 103 |
| N17 | 50261 | 71 |
| N18 | 41818 | 166 |
| N19 | 48178 | 170 |
| P11 | 53129 | 226 |
| N20 | 24646 | 172 |
| N1 | 39183 | 205 |
| N4 | 17547 | 166 |
| N2 | 29462 | 233 |
| N5 | 32392 | 45 |
| P3 | 35578 | 378 |
| N6 | 34952 | 52 |
| P1 | 10515 | 138 |
| N7 | 31143 | 64 |
| N8 | 18304 | 129 |
| N9 | 39389 | 59 |
| P4 | 20670 | 309 |
| P5 | 26335 | 214 |
| P6 | 15747 | 118 |
| N10 | 31211 | 79 |
| N11 | 14321 | 300 |
| N12 | 35918 | 180 |
| P2 | 17720 | 113 |
| N3 | 24229 | 139 |
