# Supplementary material for: Urogenital Microbiota:Potentially Important Determinant of PD-L1 Expression in Male Patients with Non-muscle Invasive Bladder Cancer
Source: BMC Microbiol. 2022 Jan 4;22:7. doi: 10.1186/s12866-021-02407-8 (PMC8725255; doi:10.1186/s12866-021-02407-8)
Supplement: Supplementary file 2 — Additional file 2: Supplementary Table S2. The information of Metadata. [file 12866_2021_2407_MOESM2_ESM.docx]

**Supplementary Table S2.The information of Metadata.**

| Sample ID | Group |
| --- | --- |
| N1 | N |
| N2 | N |
| N3 | N |
| N4 | N |
| N5 | N |
| N6 | N |
| N7 | N |
| N8 | N |
| N9 | N |
| N10 | N |
| N11 | N |
| N12 | N |
| N14 | N |
| N15 | N |
| N16 | N |
| N17 | N |
| N18 | N |
| N19 | N |
| N20 | N |
| P1 | P |
| P2 | P |
| P3 | P |
| P4 | P |
| P5 | P |
| P6 | P |
| P9 | P |
| P10 | P |
| P11 | P |
