# Supplementary figures and images for: Urogenital Microbiota:Potentially Important Determinant of PD-L1 Expression in Male Patients with Non-muscle Invasive Bladder Cancer
Source: BMC Microbiol. 2022 Jan 4;22:7. doi: 10.1186/s12866-021-02407-8 (PMC8725255; doi:10.1186/s12866-021-02407-8)

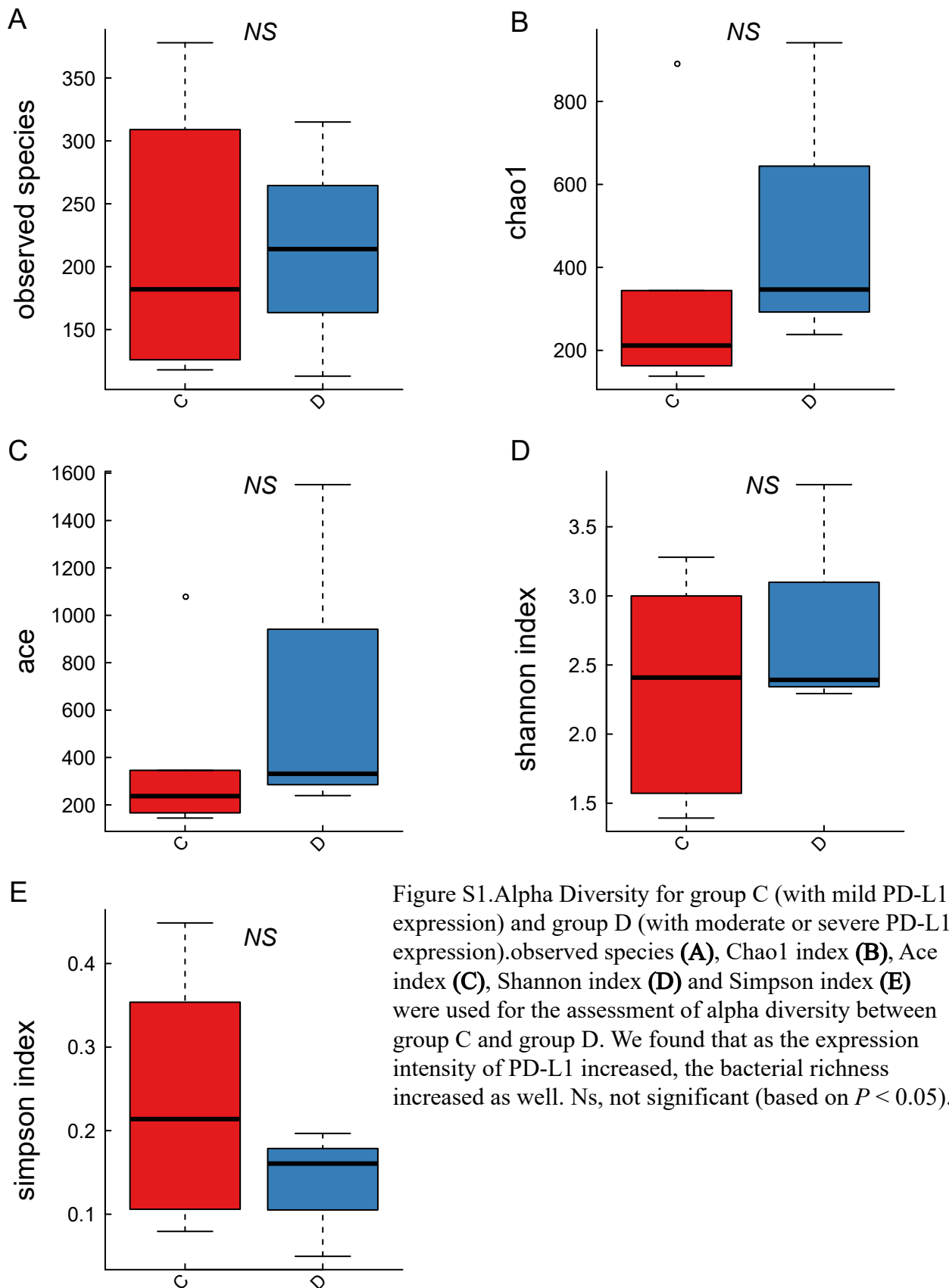

Supplement: Supplementary file 3 — Additional file 3: Figure S1. Alpha Diversity for group C (with mild PD-L1 expression) and group D (with moderate or severe PD-L1 expression).observed species (A), Chao1 index (B), Ace index (C), Shannon index (D) and Simpson index (E) were used for the assessment of alpha diversity between group C and group D. We found that as the expression intensity of PD-L1 increased, the bacterial richness increased as well. Ns, not significant (based on P < 0.05). [file 12866_2021_2407_MOESM3_ESM.pdf]
