# Supplementary material for: Urogenital Microbiota:Potentially Important Determinant of PD-L1 Expression in Male Patients with Non-muscle Invasive Bladder Cancer
Source: BMC Microbiol. 2022 Jan 4;22:7. doi: 10.1186/s12866-021-02407-8 (PMC8725255; doi:10.1186/s12866-021-02407-8)
Supplement: Supplementary file 4 — Additional file 4: Figure S2. Alpha Diversity for group F (with ≥5%tumor cells membrane staining) and group O (with < 5% tumor cells membrane staining). observed species(A),Chao1 index (B),Ace index(C),Shannon index (D) and Simpson index (E) were used for the assessment of alpha diversity between group F and group O. Urogenital microbiota of group F had higher richness than that of group O. Ns, not significant (based on P < 0.05). [file 12866_2021_2407_MOESM4_ESM.pdf]

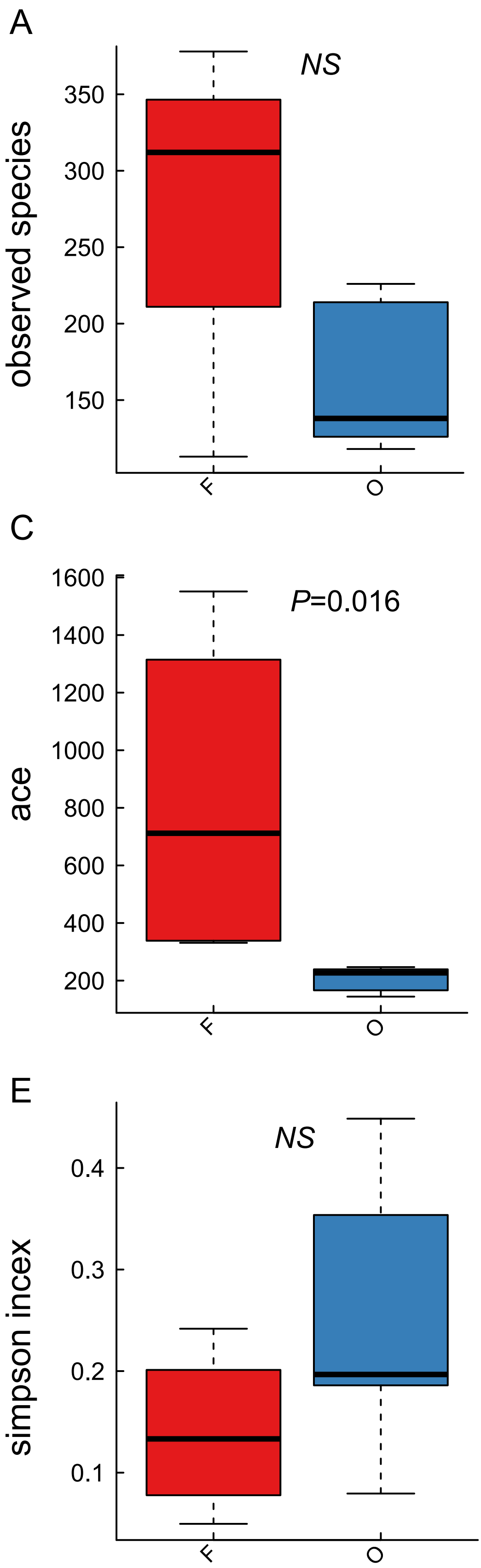

Figure S2. Alpha Diversity for group F (with ≥5% tumor cells membrane staining) and group O (with ＜5% tumor cells membrane staining). observed species(A),Chao1 index (B),Ace index(C),Shannon index (D) and Simpson index (E) were used for the assessment of alpha diversity between group F and group O. Urogenital microbiota of group F had higher richness than that of group O. Ns, not significant (based on P＜0.05).
