# Supplementary material for: Urogenital Microbiota:Potentially Important Determinant of PD-L1 Expression in Male Patients with Non-muscle Invasive Bladder Cancer
Source: BMC Microbiol. 2022 Jan 4;22:7. doi: 10.1186/s12866-021-02407-8 (PMC8725255; doi:10.1186/s12866-021-02407-8)
Supplement: Supplementary file 5 — Additional file 5: Figure S3. Specific taxa associated with different PD-L1 expression levels. Association of specific microbial taxa with higher PD-L1 expression (group F, PD-L1≥5%) and lower PD-L1 expression (group O, 1%≤PD-L1<5%) is exhibited by linear discriminant analysis effect size (LEfSe).Green indicates taxa enriched in group O and red indicates taxa enriched in group F. [file 12866_2021_2407_MOESM5_ESM.pdf]

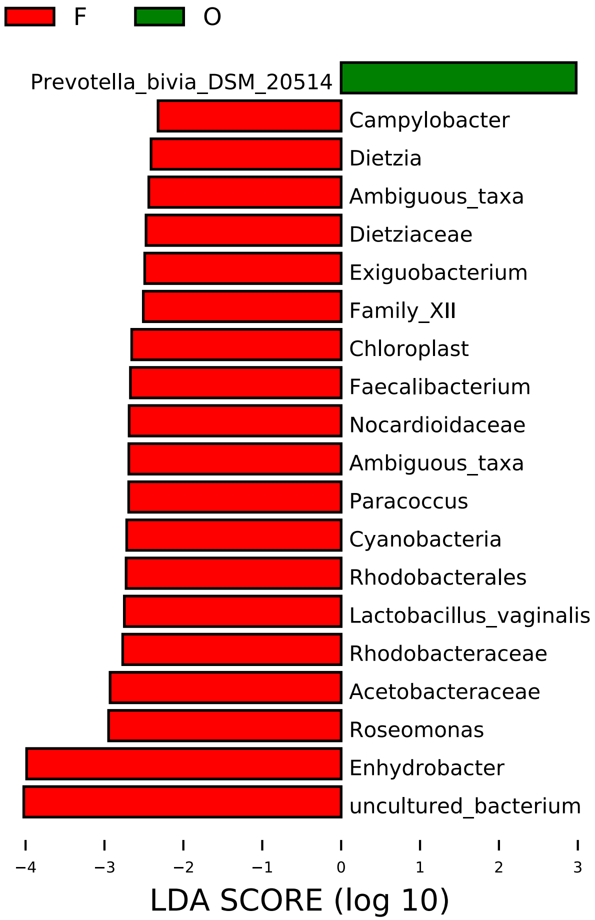

Figure S3. Specific taxa associated with different PD-L1 expression levels. Association of specific microbial taxa with higher PD-L1 expression (group F, PD-L1≥5%) and lower PD-L1 expression (group O, 1%≤PD-L1<5%) is exhibited by linear discriminant analysis effect size (LEfSe).Green indicates taxa enriched in group O and red indicates taxa enriched in group F.
